# Risk Factors for Long-term Mortality and Patterns of End-of-Life Care Among Medicare Sepsis Survivors Discharged to Home Health Care

**DOI:** 10.1001/jamanetworkopen.2020.0038

**Published:** 2020-02-26

**Authors:** Katherine R. Courtright, Lizeyka Jordan, Christopher M. Murtaugh, Yolanda Barrón, Partha Deb, Stanley Moore, Kathryn H. Bowles, Mark E. Mikkelsen

**Affiliations:** 1 Division of Pulmonary, Allergy, and Critical Care, Perelman School of Medicine, University of Pennsylvania, Philadelphia; 2 Palliative and Advanced Illness Research (PAIR) Center, Perelman School of Medicine, University of Pennsylvania, Philadelphia; 3 Center for Home Care Policy & Research, Visiting Nurse Service of New York, New York; 4 Department of Economics, Hunter College, The City University of New York (CUNY), New York; 5 National Bureau of Economic Research, Cambridge, Massachusetts; 6 Department of Biobehavioral Health Sciences, School of Nursing, University of Pennsylvania, Philadelphia

## Abstract

**Importance:**

Despite a growing recognition of the increased mortality risk among sepsis survivors, little is known about the patterns of end-of-life care among this population.

**Objective:**

To describe patterns of end-of-life care among a national sample of sepsis survivors and identify factors associated with long-term mortality risk and hospice use.

**Design, Setting, and Participants:**

This cohort study assessed sepsis survivors who were Medicare fee-for-service beneficiaries discharged to home health care using national Medicare administrative, claims, and home health assessment data from 2013 to 2014. The initial and final primary analyses were conducted in July 2017 and from July to August 2019, respectively.

**Exposures:**

Sepsis hospital discharge and 1 or more home health assessments within 1 week.

**Main Outcomes and Measures:**

Outcomes were 1-year mortality among all sepsis survivors and hospitalization in the last 30 days of life, death in an acute care hospital, and hospice use among decedents. Multivariate logistic regression was used to identify factors associated with 1-year mortality and hospice use.

**Results:**

Among 87 581 sepsis survivors who were Medicare fee-for-service beneficiaries discharged to home health care, 49 323 (56.3%) were aged 75 years or older, 69 499 (79.4%) were non-Hispanic white, and 48 472 (55.3%) were female. Among the total survivors, 24 423 (27.9%) people died within 1 year of discharge, with a median (interquartile range) survival time of 119 (51-220) days. Among these decedents, 16 684 (68.2%) were hospitalized in the last 30 days of life, 6560 (26.8%) died in an acute care hospital, and 12 573 (51.4%) were enrolled in hospice, with 5729 (45.6%) using hospice for 7 or fewer days. Several factors were associated with 1-year mortality, including a cancer diagnosis (odds ratio [OR], 3.66; 95% CI, 3.50-3.83; *P* < .001), multiple dependencies of activities of daily living or instrumental activities of daily living (OR, 2.80; 95% CI, 2.57-3.05; *P* < .001), and an overall poor health status (OR, 2.21; 95% CI, 2.01-2.44; *P* < .001) documented on home health assessment. Among the decedents, cancer was associated with hospice use (OR, 2.25; 95% CI, 2.11-2.41; *P* < .001), patients aged 85 years or older (OR, 1.49; 95% CI, 1.37-1.61; *P* < .001), and living in an assisted living setting (OR, 1.93; 95% CI, 1.69-2.19; *P* < .001).

**Conclusions and Relevance:**

The findings of this study suggest that death within 1 year after sepsis discharge may be common among Medicare beneficiaries discharged to home health care. Although 1 in 2 decedents used hospice, aggressive care near the end of life and late hospice referral were common. Readily identifiable risk factors suggest opportunities to target efforts to improve palliative and end-of-life care among high-risk sepsis survivors.

## Key Points

**Question:**

What are the risk factors for long-term mortality and patterns of end-of-life care among sepsis survivors who are Medicare beneficiaries and have been discharged to home health care?

**Findings:**

In this cohort study of 87 581 adult sepsis survivors who are Medicare fee-for-service beneficiaries and have been discharged to home health care, 1 in 4 survivors died within 1 year, and among the decedents, hospitalization and intensive care unit use in the last 30 days of life and in-hospital death were common. Several factors were found to be associated with an increased risk of mortality.

**Meaning:**

The findings of this study suggest that home health assessments may provide an opportunity to identify high-risk sepsis survivors and target efforts to improve their end-of-life care.

## Teaser

This cohort study examines the risk factors for long-term mortality and the patterns of end-of-life care among a national population of adult sepsis survivors who are Medicare fee-for-service beneficiaries and have been discharged to home health care.

## Introduction

Although sepsis is common, reductions in hospital mortality have led to an increasing number of survivors,^1,2^ with more than 1 million patients being discharged after treatment of sepsis from United States hospitals each year.^3^ However, sepsis survivorship often comes at a cost, namely reduced health-related quality of life, cognitive and functional impairments,^4^ increased risks of hospital readmission,^5,6,7^ and long-term mortality risk.^8,9,10,11^ Amid a heightened awareness of these long-term consequences,^12,13^ improving the quality of post-sepsis care has become a global priority.^13^

Efforts to improve outcomes among sepsis survivors have largely focused on preventing or mitigating postdischarge morbidity and mortality. Nearly 1 in 2 sepsis survivors receive postacute care services, such as home health care, inpatient rehabilitation, and skilled nursing facility placement.^5,14,15^ Yet, the long-term risk of death after sepsis remains high compared with hospitalized patients without sepsis.^7,9,16^ Despite mounting evidence that sepsis survivorship is associated with increased mortality, little attention has been paid to the patterns of end-of-life care among this population. This is a particularly important evidence gap to fill in sepsis research considering the sustained national focus on improving the quality of end-of-life care for all seriously ill adults.^17,18^ Furthermore, as policy recommendations regarding optimal advance care planning practice and use of community-based palliative care services continue to evolve,^19,20,21,22^ it is important for sepsis survivors to be recognized as a potential population in need of these services.

In this national study, we assessed the risk of long-term mortality and end-of-life care among Medicare beneficiaries discharged to home health care after sepsis. Homes are common postacute care destinations after sepsis, second only to skilled care facilities.^5,14,15^ Annually, approximately 200 000 sepsis survivors are discharged to home to receive health care services such as skilled nursing, physical and occupational therapy, and health aid visits.^15^ Because home health care spending is projected to outpace any other national health expenditure over the next decade,^14^ it is essential to evaluate outcomes among this population. In the present study, 1-year mortality rates were assessed and factors associated with mortality were identified from the sepsis hospitalization and the initial home health assessment, including functional assessments. The rates of hospitalization among the decedents in the last 30 days of life, in-hospital death, and hospice use, were assessed and characteristics associated with hospice use were identified.

## Methods

### Data Sources and Study Population

This retrospective cohort study used Medicare administrative and claims files from calendar years 2013 and 2014 to identify patients with sepsis who were hospitalized and discharged to home health care between July 1, 2013, and December 31, 2013, and to evaluate hospice and mortality outcomes up to 1 year after hospital discharge. The files used included the Medicare Beneficiary Summary file, Medicare Inpatient Standard Analytic File (SAF), Outpatient SAF, Home Health SAF, Hospice SAF, Part B SAF, Chronic Conditions Warehouse (CCW), and US Census data.^52^ We linked these files with the Outcome and Assessment Information Set (OASIS)–C,^23,24^ a comprehensive, federally mandated assessment of patients’ health, social, cognitive, and functional status, which is completed on initiation of home health care services. This study was approved by the respective institutional review boards of the Visiting Nurse Service of New York and the University of Pennsylvania, with a waiver of informed consent under category 4 of the Federal Policy for the Protection of Human Subjects (45 CFR 46) in 2015. This report followed the Strengthening the Reporting of Observational Studies in Epidemiology (STROBE) reporting guideline.^25^ Initial and final primary analyses were conducted in July 2017 and July and August 2019, respectively.

Sepsis was defined using a combination of 2 strategies given the limited sensitivity of sepsis identification from administrative claims.^26^ First, we used the *International Classification of Diseases and Related Health Problems, Ninth Revision, Clinical Modification (ICD-9-CM)* codes 995.91 (sepsis without organ dysfunction), 995.92 (severe sepsis), and 785.52 (septic shock), which were added to the *ICD-9-CM* codes in 2003 to improve accuracy of case identification.^26^ Second, we used the implicit approach developed by Angus and colleagues^27^ that requires an ICD-9-CM code for infection and end-organ dysfunction, which was initially developed using 1995 claims data and was subsequently validated using administrative claims from 2009 to 2010^28^ and medical records from 2005 to 2009.^26^

Beneficiaries had to be at least 18 years of age and have received at least 1 home health care visit within 1 week of discharge. Beneficiaries with additional health care use (hospital readmission, observation unit stay, and hospice admission) before their first home health visit and those without complete OASIS-C data were excluded from the study. Finally, we included only the index sepsis discharge to home health care in this sample to ensure independence of observations.

### Outcomes

The 1-year mortality among all sepsis survivors and hospice use among decedents were examined. The date of death was obtained from Medicare records and time to death was calculated from the discharge date of index hospital stay. The Inpatient SAF was used to identify hospitalization and intensive care unit (ICU) use within the last 30 days of life and acute care hospital as site of death. Hospice enrollment and length of stay were identified using the Hospice SAF, with late hospice referral defined as a hospice admission date 7 or fewer days prior to the date of death.^29,30,31^

### Individual Characteristics

Medicare administrative data provided patients’ demographic characteristics, comorbidities, and clinical characteristics from the index sepsis discharge, including the admission type (Medicare Severity–Diagnosis Related Group [MS-DRG]), ICU use, and infection source. To address underreporting in claims data, ethnicity and median family income in the county where the patient lived were obtained from OASIS-C and census data, respectively, and a diagnosis of Alzheimer disease and associated dementias was obtained from the CCW.

The initial OASIS-C assessment was conducted by a trained home health clinician at the start of a new episode of home health care within 2 days of hospital discharge for 81% of the cohort (n = 70 941) and within 7 days for the remaining sample (n = 16 640). More than 100 items were assessed, including activities of daily living (ADLs), instrumental activities of daily living (IADLs), living arrangements, cognitive functioning, sensory and behavioral status, disease signs and symptoms by organ system, frailty, and overall health status.^32,33^

### Statistical Analyses

Variables were summarized using frequencies and proportions for categorical data or means (SDs) and medians (interquartile ranges [IQRs]) for continuous data. Bivariate analyses were performed using a χ^2^ test to compare patient characteristics between decedents and survivors. Survival data were expressed as medians (IQRs), and analyses were performed using the Kaplan-Meier method, with censoring of all patients who remained alive 365 days after sepsis discharge.

Two multivariate logistic regression models were built using forward selection to examine the independent associations between patient characteristics and 1-year mortality among all sepsis survivors and hospice use among the decedents. Candidate covariates were selected a priori based on existing literature and clinical expertise and included those variables with a 2-sided *P* < .05 in the final multivariate models. Variance inflation factor diagnostic tests were used to check for collinearity between covariates and those with a value greater than 10 were excluded from the final models.^34^ Both final models included the following characteristics known before and during the index sepsis hospitalization: age, race/ethnicity, Medicaid status, comorbidities,^35^ sepsis severity, hospital-acquired sepsis, infection source, ICU admission, and surgical admission type. Items from the postdischarge OASIS-C home health assessment included in the final models are provided in Table 1 and include the following: risk for hospitalization, overall health status, living arrangement, impaired vision, number of medications, dyspnea, cognitive impairment, and the number of ADL or IADL dependencies. Categorical variables were collapsed in the final models based on the distribution of responses. Data completeness was excellent; 168 patients (1.9%) with an unknown or unclear or missing response for the overall status item on the home health assessment were excluded from the models.

**Table 1.** Characteristics of 87 581 Patients in the Medicare Sepsis Survivor Cohort Discharged to Home Health Care

Characteristic^a^	No. (%) of Patients
Age, y	
<65^b^	14 236 (16.3)
65-74	24 022 (27.4)
75-84	28 067 (32.0)
≥85	21 256 (24.3)
Race	
Black	10 744 (12.3)
Non-Hispanic white	69 499 (79.4)
Hispanic	4773 (5.5)
Other^c^	2565 (2.9)
Sex	
Female	48 472 (55.3)
Male	39 109 (44.7)
Medicaid eligible	23 381 (26.7)
Annual family income, $, mean (SD)	54 100 (14 810)
Comorbidities	
Hypertension	60 474 (69.0)
Fluid and electrolyte disorders	48 439 (55.3)
Deficiency anemias	31 687 (36.2)
Renal failure	29 802 (34.0)
Alzheimer disease and related disorders	28 095 (32.1)
Chronic pulmonary disease	28 153 (32.1)
Congestive heart failure	21 479 (24.5)
Diabetes without chronic complications	24 943 (28.5)
Metastatic cancer, lymphoma, or solid tumor without metastasis^d^	11 427 (13.0)
Hypothyroidism	17 340 (19.8)
Coagulopathy	13 534 (15.5)
Weight loss	9693 (11.1)
Other neurological disorders	11 958 (13.7)
Peripheral vascular disease	10 469 (12.0)
Depression	11 950 (13.6)
Obesity	13 171 (15.0)
Diabetes with chronic complications	9672 (11.0)
Total No. of comorbidities, mean (SD)	4.35 (1.89)
Infection source	
Kidney, urinary tract, and other genitourinary	36 202 (41.3)
Pneumonia and other respiratory	30 666 (35.0)
Bone, joint, and skin/soft tissue	13 458 (15.4)
Gastrointestinal	8721 (9.96)
Device related	3264 (3.73)
Bacteremia	1992 (2.27)
Cardiovascular/endocarditis	1263 (1.44)
Postoperative	1905 (2.18)
Central nervous system	331 (0.38)
Other or unknown	24 959 (28.5)
Sepsis severity	
Sepsis	11 934 (13.6)
Severe sepsis	70 513 (80.5)
Septic shock	5134 (5.86)
Intensive or cardiac care unit admission	44 241 (50.5)
Medical admission type	70 135 (80.1)
Home health assessment	
Indicators for risk of hospitalization^b^	
Decline in mental, emotional, behavioral condition	13 425 (15.3)
Multiple hospitalizations (≥2 in past 12 mo)	43 993 (50.2)
Frailty indicators (weight loss, self-reported exhaustion)	36 127 (41.2)
Assessment of overall health status	
Stable, no risks of complications or death	4248 (4.9)
Temporarily high risks but likely to return to stable condition	42 081 (48.0)
Fragile health/ongoing risks of complications/death	33 950 (38.8)
Serious progressive conditions that could lead to death within 1 y	7134 (8.2)
Unknown or missing	168 (0.2)
Vision	
Normal	17 529 (71.6)
Partially impaired	6297 (25.7)
Severely impaired	652 (2.7)
Speech and oral expression	
Expresses complex ideas/feelings/needs with no impairment	50 830 (58.0)
Minimal difficulty in expressing ideas and needs	26 481 (30.2)
Expresses simple ideas/needs with moderate difficulty	6163 (7.0)
Severe difficulty expressing simple ideas/needs	2326 (2.7)
Unable to express basic needs but not comatose or unresponsive	1045 (1.2)
Patient nonresponsive or unable to speak	736 (0.8)
Frequency of pain interfering with activity	
No pain	23 107 (26.4)
Pain that does not interfere with activity or movement	7814 (8.9)
Less often than daily	10 148 (11.6)
Daily, but not constantly	35 611 (40.7)
All the time	10 901 (12.4)
Living arrangements	
Lives alone	16 404 (18.7)
Lives with someone	66 098 (75.5)
Lives in congregate (eg, assisted living)	5079 (5.8)
Presence of dyspnea	
Not short of breath	19 056 (21.8)
Walking >20 ft, climbing stairs	18 467 (21.1)
With moderate exertion	28 987 (33.1)
With minimal exertion	16 193 (18.5)
At rest (during day or night)	4878 (5.6)
Respiratory treatments needed	23 791 (27.2)
Cognitive function	
Alert and/or oriented	46 049 (52.6)
Requires prompting	27 893 (31.8)
Requires assistance or direction	8974 (10.2)
Requires considerable assistance or direction	3422 (3.9)
Totally dependent	1243 (1.4)
No. of ADL or IADL dependencies	
0-2	10 710 (12.2)
3-5	22 317 (25.5)
6-8	22 501 (25.7)
9-11	24 492 (28.0)
12-13	7561 (8.6)

Abbreviations: ADL, activities of daily living; IADL, instrumental ADLs.

^a^ Percentages in each category may not add up to 100 owing to rounding.

^b^ Of the 14 236 Medicare beneficiaries aged younger than 65 years, 12 145 (85.3%) received disability insurance benefits, 600 (4.2%) received end-stage renal disease benefits, and 1458 (10.2%) received disability and end-stage renal disease benefits.

^c^ Other includes mixed race/ethnicity or not stated.

^d^ Includes patients with cancer diagnoses present within the 6 months prior to sepsis discharge date.

In secondary analyses, we included only covariates with a bivariate association of *P* < .001 to avoid overfitting the models. The results of these parsimonious models remained unchanged; thus, the results of the full explanatory models are presented. Analyses stratified by the presence of a cancer diagnosis, which has previously been shown to be associated with in-hospital mortality and hospice referral among sepsis readmissions, were also performed.^8^

For all analyses, given the large sample size, statistical testing was 2-sided with a significance threshold of *P* < .001, and results were preferentially judged by their clinical relevance. RStudio, version 3.3.1 (R Core Team), was used for descriptive statistics and statistical analyses. SAS, version 9.4 (SAS Institute Inc) was used for diagnostic variance inflation factor tests and Kaplan-Meier plot generation.

## Results

**Figure 1.**
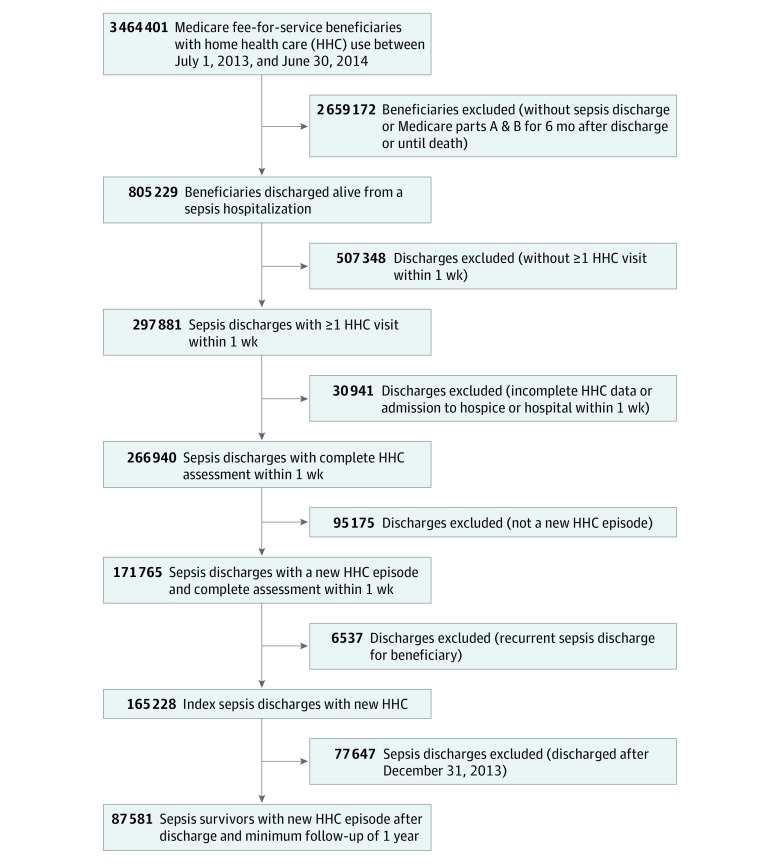
Flow Diagram for Identification of Cohort of Medicare Sepsis Survivors Discharged to Home Health Care

The cohort included 87 581 sepsis survivors with Medicare insurance who were discharged to new home health care services between July 1, 2013, and December 31, 2013 (Figure 1). Among them, 49 323 (56.3%) patients were aged 75 years or older, 69 499 (79.4%) were non-Hispanic white, and 48 472 (55.3%) were women. Overall, patients had a mean (SD) total number of comorbidities of 4.35 (1.89), and 64 200 (73.3%) were not Medicaid-eligible (Table 1). Severe sepsis was observed in most cases (70 513 [80.5%]), with the genitourinary system being the most common infection source (36 202 [41.3%]), and only half of the patients (44 241 [50.5%]) having received care in an ICU during the index sepsis hospitalization. Home health care assessments after discharge suggested that half of the patients had at least 1 indicator of risk for hospitalization (43 993 [50.2%]) and dependency in more than 2 ADLs/IADLs (76 871 [87.8%]). Uncontrolled symptoms were common, with 46 512 (51.3%) of patients experiencing pain daily or constantly and 68 525 (78.3%) having dyspnea on exertion or at rest. Nearly half of the patients had at least mild limitations in cognitive function (41 532 [47.3%]) and speech (36 751 [41.9%]).

Nursing visits, which were received by 83 537 [95.4%] patients, were the most common home health care service provided within 7 days of sepsis discharge, followed by physical therapy (46 096 [52.6%]), occupational therapy (14 114 [16.1%]), and speech therapy (1897 [2.2%]). During the first week, 34 064 (38.9%) sepsis survivors were seen in the ambulatory setting by a medical professional.

### Mortality and End-of-Life Health Care Use

**Figure 2.**
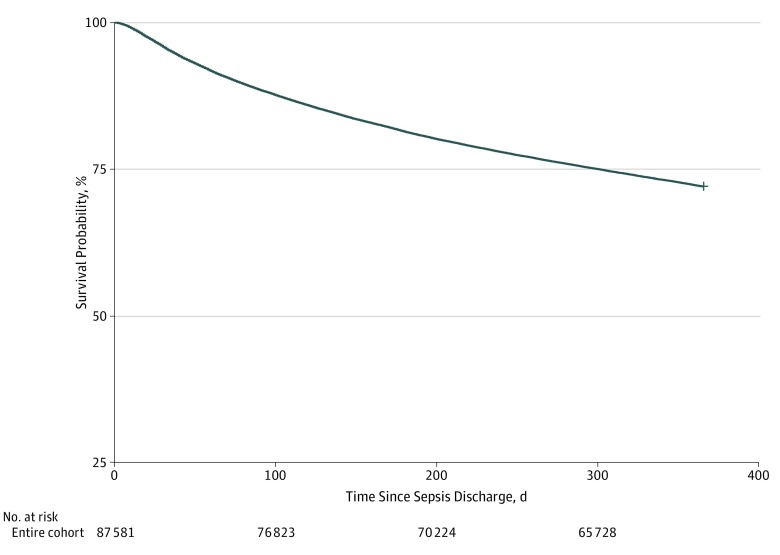
One-Year Survival Among 87 581 Patients After Sepsis Discharge to Home Health Care

Among the total survivors, 24 423 (27.9%) patients died within 1 year of discharge, with a median (IQR) survival time of 119 (51-220) days (Figure 2). The characteristics of decedents and survivors at 1 year after discharge differed in several patient-related, sepsis-related, and home health assessment characteristics (eTables 1 and 2 in the Supplement), most notably in the proportion of patients with a cancer diagnosis (decedents, 5965 [24.4%] vs survivors, 5462 [8.2%]; *P* < .001). Among all decedents, 16 684 (68.2%) were hospitalized, 10 190 (61.1%) were admitted to an ICU during the last 30 days of life, and 6560 (26.8%) died in an acute care hospital (Table 2). In total, 12 573 (51.4%) decedents were enrolled in hospice prior to death, with a median (IQR) time from sepsis discharge to hospice enrollment of 100 (36-199) days. The median (IQR) hospice length of stay was 10 (3-33) days, with 5729 (45.6%) of those enrolled receiving hospice services for 7 or fewer days prior to death.

**Table 2.** Acute Care and Hospice Use Among 24 423 Decedents

Acute Care in Last 30 d of Life	No. (%) of Patients
Hospitalization	16 684 (68.2)
Intensive/cardiac care unit admission	10 190 (61.1)
Death in acute care hospital	6560 (26.8)
Hospice	
Any hospice use	12 573 (51.4)
Days to enrollment from sepsis discharge, median (IQR)	100 (36-199)
Length of stay, median (IQR), d	10 (3-33)
Use ≤7 d	5729 (45.6)

Abbreviation: IQR, interquartile range.

### Factors Independently Associated With Risk of 1-Year Mortality

In multivariate analyses, several factors were found to be independently associated with an increased risk of 1-year mortality (Table 3). Patient-level risk factors included older age (≥85 years, OR, 1.47; 95% CI, 1.40-1.54; *P* < .001) and the presence of comorbid conditions in general and cancer diagnosis in particular (OR, 3.66; 95% CI, 3.50-3.83; *P* < .001). Sepsis-related factors known at the time of discharge that were independently associated with an increased risk of 1-year mortality included severe sepsis (OR, 1.30; 95% CI, 1.23-1.37; *P* < .001), pneumonia and other respiratory infection source (OR, 1.14; 95% CI, 1.09-1.18; *P* < .001), and ICU use (OR, 1.07; 95% CI, 1.03-1.11; *P* < .001). Characteristics that appeared to be protective against mortality within the year after sepsis were age younger than 65 years, female sex, obesity, hypertension, and postoperative infection, or a surgical admission during the index sepsis admission.

**Table 3.** Multivariate Regression Results for 1-Year Mortality Among All Sepsis Survivors and Hospice Enrollment Among Decedents

Characteristic	Death Within 1 y of Sepsis Discharge, OR (95% CI) (n = 87 413)	*P* Value	Hospice Enrollment Among Decedents, OR (95% CI) (n = 24 423)	*P* Value
**Demographic**				
Age, y				
65-74	1 [Reference]		1 [Reference]	
<65	0.84 (0.79-0.89)	<.001	0.76 (0.69-0.84)	<.001
75-84	1.08 (1.03-1.13)	<.001	1.23 (1.14-1.32)	<.001
≥85	1.47 (1.40-1.54)	<.001	1.49 (1.37-1.61)	<.001
Race/ethnicity				
Non-Hispanic white	1 [Reference]		1 [Reference]	
Black	1.04 (0.98-1.09)	.12	0.67 (0.61-0.73)	<.001
Hispanic	0.91 (0.84-0.98)	.01	0.81 (0.71-0.92)	.001
Other	0.84 (0.76-0.92)	<.001	0.64 (0.54-0.76)	<.001
Sex				
Female	0.85 (0.82-0.87)	<.001	1.11 (1.05-1.17)	<.001
Medicaid eligible	0.94 (0.90-0.98)	.005	0.77 (0.72-0.82)	<.001
**Comorbidities**				
Metastatic cancer, lymphoma, solid tumor without metastasis	3.66 (3.50-3.83)	<.001	2.25 (2.11-2.41)	<.001
Hypertension	0.87 (0.84-0.90)	<.001	0.95 (0.89-1.00)	.06
Fluid and electrolyte disorders	1.03 (1.00-1.07)	.05	0.99 (0.94-1.05)	.76
Deficiency anemias	1.18 (1.14-1.22)	<.001	1.02 (0.97-1.08)	.48
Renal failure	1.37 (1.32-1.42)	<.001	0.94 (0.88-0.99)	.02
Chronic pulmonary disease	1.18 (1.14-1.23)	<.001	0.89 (0.84-0.95)	<.001
Diabetes	0.99 (0.96-1.03)	.63	0.90 (0.85-0.96)	<.001
Congestive heart failure	1.35 (1.30-1.40)	<.001	0.85 (0.80-0.90)	<.001
Obesity	0.76 (0.69-0.76)	<.001	0.84 (0.76-0.92)	<.001
Depression	0.93 (0.89-0.98)	.003	1.13 (1.04-1.23)	.003
Peripheral vascular disease	1.20 (1.14-1.26)	<.001	0.84 (0.77-0.91)	<.001
Weight loss	1.50 (1.43-1.58)	<.001	1.06 (0.98-1.14)	.15
Alzheimer disease and associated disorders	1.13 (1.09-1.18)	<.001	1.09 (1.02-1.16)	.009
**Index Sepsis Discharge**				
Sepsis infection source				
Bone/joint/skin/tissue	1.03 (0.98-1.08)	.30	0.78 (0.72-0.85)	<.001
Central nervous system	0.71 (0.51-0.97)	.03	0.75 (0.41-1.35)	.33
Pneumonia and other respiratory	1.14 (1.09-1.18)	<.001	0.94 (0.89-1.00)	.04
Postoperative	0.58 (0.51-0.67)	<.001	1.02 (0.79-1.31)	.89
Sepsis severity				
Sepsis	1 [Reference]		1 [Reference]	
Severe sepsis	1.30 (1.23-1.37)	<.001	1.04 (0.96-1.14)	.35
Septic shock	1.14 (1.05-1.24)	.003	0.88 (0.76-1.01)	.06
Intensive or cardiac care unit admission	1.07 (1.03-1.11)	<.001	0.91 (0.86-0.96)	<.001
Surgical admission type	0.70 (0.67-0.73)	<.001	0.90 (0.83-0.98)	.01
**Home Health Assessment Within 7 d of Sepsis Discharge**				
Indicators for risk of hospitalization on home health assessment				
No risks	1 [Reference]		1 [Reference]	
Decline in mental, emotional, or behavioral condition	0.99 (0.95-1.04)	.63	1.05 (0.98-1.13)	.18
Multiple hospitalizations (≥2 in past 12 mo)	1.21 (1.17-1.26)	<.001	1.01 (0.96-1.07)	.75
Frailty indicators (weight loss, self-reported exhaustion)	1.07 (1.03-1.11)	<.001	1.04 (0.98-1.10)	.19
Assessment of overall health status^a^				
No risk	1 [Reference]		1 [Reference]	
Temporarily high risks but likely to return to stable condition	0.96 (0.88-1.04)	.35	1.02 (0.88-1.19)	.76
Fragile health/ongoing risks of complications or death	1.39 (1.27-1.51)	<.001	1.23 (1.06-1.43)	.008
Serious progressive conditions that could lead to death within 1 y	2.21 (2.01-2.44)	<.001	1.40 (1.19-1.65)	<.001
Vision				
No impairment	1 [Reference]		1 [Reference]	
Partially impaired	0.96 (0.93-1.00)	.81	0.98 (0.92-1.05)	.57
Severely impaired	0.97 (0.87-1.08)	.61	1.07 (0.90-1.27)	.46
Speech and oral expression				
No impairment	1 [Reference]		1 [Reference]	
Minimal impairment	1.02 (0.98-1.07)	.34	1.01 (0.94-1.09)	.78
Moderate to severe impairment	1.09 (1.02-1.17)	.01	1.11 (0.99-1.24)	.07
Unable to speak	1.13 (0.99-1.30)	.07	0.94 (0.76-1.15)	.53
Cognitive function				
No impairment	1 [Reference]		1 [Reference]	
Mild	0.99 (0.95-1.04)	.78	1.07 (1.00-1.15)	.06
Moderate	1.15 (1.04-1.26)	.006	1.14 (0.98-1.32)	.09
Severe	1.08 (0.92-1.27)	.35	0.78 (0.62-0.98)	.04
Living arrangements				
Lives alone	1 [Reference]		1 [Reference]	
Lives with someone	1.07 (1.02-1.12)	.003	1.16 (1.08-1.26)	<.001
Lives in congregate (eg, assisted living)	1.21 (1.12-1.31)	<.001	1.93 (1.69-2.19)	<.001
ADL and/or IADL dependencies				
0-2	1 [Reference]		1 [Reference]	
3-5	1.12 (1.05-1.19)	<.001	1.18 (1.05-1.33)	.005
6-8	1.34 (1.25-1.43)	<.001	1.16 (1.04-1.31)	.01
9-11	1.90 (1.78-2.03)	<.001	1.22 (1.08-1.37)	.001
12-13	2.80 (2.57-3.05)	<.001	1.15 (0.99-1.32)	<.07
Presence of dyspnea				
Not short of breath	1 [Reference]		1 [Reference]	
With moderate exertion/walking >20 ft, climbing stairs	1.10 (1.05-1.16)	.02	0.94 (0.87-1.03)	.21
With minimal exertion	1.30 (1.23-1.37)	<.001	0.95 (0.86-1.04)	.02
At rest (during day or night)	1.53 (1.42-1.66)	<.001	0.87 (0.78-0.99)	.03
Frequency of pain interfering with activity				
No pain	1 [Reference]		1 [Reference]	
Sometimes	0.90 (0.86-0.95)	<.001	1.00 (0.92-1.08)	.94
Often	0.90 (0.86-0.94)	<.001	0.98 (0.92-1.05)	.59
Log likelihood	−45 212.91		−15 825.15	
Akaike information criterion	90 537.81		31 762.31	

Abbreviations: ADL, activities of daily living; IADL, instrumental ADLs; OR, odds ratio.

^a^ Patients with an “unknown or unclear” or missing response for overall health status were excluded from regression analyses (n = 168).

The initial home health care assessment identified several additional factors that were independently associated with an increased risk of 1-year mortality, including dependence in multiple ADLs/IADLs (OR, 2.80; 95% CI, 2.57-3.05; *P* < .001), dyspnea at rest (OR, 1.53; 95% CI, 1.42-1.66; *P* < .001), 2 or more hospitalizations in the past 12 months (OR, 1.21; 95% CI, 1.17-1.26; *P* < .001), frailty (OR, 1.07; 95% CI, 1.03-1.11; *P* < .001), living in an assisted living setting (OR, 1.21; 95% CI, 1.12-1.31; *P* < .001), and an overall poor health status (OR, 2.21; 95% CI, 2.01-2.44; *P* < .001).

### Factors Independently Associated With Hospice Use

Several sociodemographic and clinical factors were independently associated with higher odds of receiving hospice care prior to death, including older age (≥75 years: OR, 1.23; 95% CI, 1.14-1.32; ≥85 years: OR, 1.49; 95% CI, 1.37-1.61; *P* < .001), female sex (OR, 1.11; 95% CI, 1.05-1.17; *P* < .001), and a cancer diagnosis (OR, 2.25; 95% CI, 2.11-2.41; *P* < .001). Similar to the mortality model, the postdischarge initial home health assessment identified additional factors independently associated with increased odds of hospice use independent of patient-related and sepsis-related factors, including an overall poor health status (OR, 1.40; 95% CI, 1.19-1.65 *P* < .001) and living with someone (OR, 1.16; 95% CI, 1.08-1.26; *P* < .001) or in an assisted living setting (OR, 1.93; 95% CI, 1.69-2.19; *P* < .001) (Table 3). Of note, multiple factors were independently associated with significantly reduced odds of hospice use, including age younger than 65 years (OR, 0.76; 95% CI, 0.69-0.84; *P* < .001), non-Hispanic white race (OR, 0.77; 95% CI, 0.72-0.82), being Medicaid-eligible (OR, 0.77; 95% CI, 0.72-0.82; *P* < .001), noncancer comorbidities (chronic pulmonary disease, heart failure, diabetes, obesity, and peripheral vascular disease), ICU use (OR, 0.91; 95% CI, 0.86-0.96; *P* < .001), or a bone/joint/skin/tissue infection source (OR, 0.78; 95% CI, 0.72-0.85; *P* < .001) during the index sepsis stay.

### Role of Cancer in Mortality and Hospice Use After Sepsis

In this cohort, 11 427 (13.0%) sepsis survivors discharged to home health care services had a cancer diagnosis. Among them, older age (≥85 years: OR, 1.09; 95% CI, 0.95-1.23; *P* = .21) and the source and severity of sepsis were no longer significantly associated with 1-year mortality. Of note, dependence in multiple ADLs/IADLs (OR, 2.55; 95% CI, 2.01-3.23; *P* < .001) and an overall poor health status (OR, 2.98; 95% CI, 2.33-3.80; *P* < .001) after discharge remained independently associated. Among the 5965 (24.4%) decedents with cancer, age and sex were no longer associated with hospice use. Complete results from stratified analyses are given in eTables 3 and 4 in the Supplement.

## Discussion

In this national cohort study, we examined risk factors for long-term mortality and patterns of end-of-life care among sepsis survivors who were Medicare beneficiaries and were discharged to home health care. More than one-quarter of patients died in the year following discharge, with most deaths occurring within 6 months. Two-thirds of the decedents were admitted to a hospital in the last 30 days of life where more than half received care in an ICU and 1 in 4 died. We also identified several patient-related, sepsis-related, and home health assessment factors associated with mortality and hospice use after sepsis survivorship.

The 1-year mortality rate among sepsis survivors in the present study is within the range previously reported in the literature.^9,16,36^ Shankar-Hari et al^16^ and Yende et al^36^ recently reported 1-year mortality rates of 15% and 17.6%, respectively, among relatively young and previously healthy patients with few comorbidities and high rates of prehospital functional independence. In contrast, Prescott et al^9^ found a 1-year mortality rate of 48.5% among sepsis survivors in the Health and Retirement Study cohort, which includes an older population with multiple comorbidities and at least some functional dependence at baseline. The most likely explanation for such a wide range in mortality rates is the variation in the populations studied. Although patterns over time in the risk of long-term mortality after sepsis have not been described, if the pattern follows the decline seen in acute sepsis mortality over the past decade,^2^ the more contemporary data used in this study may further explain the lower mortality rate observed compared with that observed by Prescott et al.^9^ Finally, the present study focused on the Medicare home health care population, which tends to be older and sicker and more likely to live below the federal poverty level compared with general Medicare beneficiaries.^37^ However, whether and how much home health care is associated with long-term sepsis survivorship remains unknown and warrants further study.

The present study’s results support previous reports of older age, male sex, medical admission type, comorbidities, and cancer in particular, being important risk factors for long-term mortality after sepsis.^16,38^ Additional sepsis-related risk factors were identified, including severe sepsis, respiratory infection source, and ICU use. Such information may be useful to guide future efforts to develop and test risk stratification models among sepsis survivors, which may facilitate tailoring postdischarge care decisions. For example, the optimal intensity and timing of postacute care services^39^ and follow-up with primary care clinicians^40^ for sepsis survivors is unknown.

Several factors were identified on the home health care assessment after discharge that were associated with death within 1 year, independent of the foregoing patient-related and sepsis-related factors. Such factors included dyspnea at rest, 2 or more hospitalizations in the past 12 months, living in an assisted living setting, and an overall poor health status. In addition, similar to recent findings among Medicare sepsis survivors discharged to a skilled nursing facility,^41^ dependence in multiple ADLs was an independent risk factor for mortality after sepsis in this home health care population. Moreover, we found a high prevalence of uncontrolled pain and dyspnea after discharge, 2 of the most common reasons for emergency department visits and readmissions among chronically ill patients.^42,43^ These findings suggest that there is a unique opportunity for trained home health care clinicians to identify high-risk sepsis survivors and facilitate targeted interventions. For example, such patients may benefit from more frequent contact with their primary care or specialty clinicians, advance care planning, palliative care consultation, or even hospice referral in some cases. Patients referred to hospice from nonhospital sources are more likely to receive end-of-life care consistent with the preferences of most patients in the United States facing serious illness, including continuous home hospice care and dying at home.^29,44^

Among the decedents in this cohort, we found that approximately two-thirds were admitted to the ICU in the last 30 days of life, which is a rate nearly 3 times that recently reported among all Medicare fee-for-service beneficiaries during a similar time frame.^45^ Furthermore, ICU use during the index sepsis stay was associated with lower odds of subsequent hospice use prior to death despite being associated with significantly increased risk of mortality. Although this observational study could not determine causality, we believe that prior ICU use as a potential barrier to hospice enrollment is an important area for further exploration. It is possible that for patients who recently survived a sepsis hospitalization and often an ICU stay, patients, families, and clinicians alike may rely on that past performance to predict the future.^46^ Such performance heuristics are common in medical decision-making, and often serve as a barrier to seeing the overall trajectory of functional decline that is common among patients with chronic illness.^47^

Although the rate of hospice enrollment among the decedents in this home health sepsis cohort was similar to a recent report among a general Medicare fee-for-service population,^45^ the median hospice length of stay in the present study was considerably shorter. This finding suggests a missed opportunity within home health care to improve end-of-life care, supported by the finding that the median time to hospice admission was 100 days from sepsis discharge. For example, patients with a diagnosis of heart failure, chronic pulmonary disease, or peripheral vascular disease in this cohort had considerably increased mortality risk, yet they were less likely to receive hospice care. Thus, earlier disease-specific interventions to improve end-of-life care in this population may be needed.

### Limitations

This study has limitations. First, although this study offers an important first look at mortality risk and end-of-life care outcomes among sepsis survivors discharged to home health care, these results may not be generalizable to other sepsis populations, such as those discharged to home without home health care or those discharged to institutional postacute care. Prospective observational studies, designed to confirm whether functional and overall health status assessment are associated with mortality among sepsis survivors discharged to home, are needed. Second, given the inherent limitations of identifying sepsis survivors using administrative claims, future studies may benefit from sepsis identification from electronic clinical records in accordance with current international sepsis definitions.^48^ Third, a comparison group of nonsepsis hospital discharges to home health care was not available to quantify how many of the present study’s findings are directly attributable to sepsis vs other diagnoses with home health care. We were also unable to examine in this retrospective study whether the outcomes were mediated by types of home health care services received owing to unmeasurable indication bias. Prior studies have found mixed evidence for the efficacy of physical or occupational therapy after sepsis^49,50^; consequently, pragmatic randomized trials are needed. In addition, we acknowledge that patient preferences regarding end-of-life care were unknown for this cohort such that it was not possible to assess whether hospitalization near the end of life or dying in the hospital reflected goal-concordant care in some cases.^51^

## Conclusions

Improvements in sepsis care have led to an increase in short-term survival, yet long-term mortality rates after hospital discharge remain high. Many sepsis survivors have readily identifiable characteristics that are associated with an increased risk of death, which may help direct interventions that mitigate the high rates of aggressive and intensive care experienced near the end of life among this population. Further research is needed to understand the association of postacute care services with mortality risk and end-of-life outcomes among sepsis survivors.

## Notes

Supplement. **eTable 1.** Characteristics of Medicare Sepsis Survivor Cohort Discharged to Home Health Care by 1-Year Survival Status **eTable 2.** Home Health Assessment Within 1 Week After Sepsis Discharge Among Medicare Beneficiaries by 1-Year Survival Status **eTable 3.** Multivariate Regression Results for 1-Year Mortality Among All Sepsis Survivors Stratified by Presence of Cancer **eTable 4.** Multivariate Regression Results for Hospice Enrollment Among Decedents Stratified by Presence of Cancer
