# Supplementary material for: Risk Factors for Long-term Mortality and Patterns of End-of-Life Care Among Medicare Sepsis Survivors Discharged to Home Health Care
Source: JAMA Netw Open. 2020 Feb 26;3(2):e200038. doi: 10.1001/jamanetworkopen.2020.0038 (PMC7137683; doi:10.1001/jamanetworkopen.2020.0038)
Supplement: Supplement. — eTable 1. Characteristics of Medicare Sepsis Survivor Cohort Discharged to Home Health Care by 1-Year Survival Status eTable 2. Home Health Assessment Within 1 Week After Sepsis Discharge Among Medicare Beneficiaries by 1-Year Survival Status eTable 3. Multivariate Regression Results for 1-Year Mortality Among All Sepsis Survivors Stratified by Presence of Cancer eTable 4. Multivariate Regression Results for Hospice Enrollment Among Decedents Stratified by Presence of Cancer [file jamanetwopen-3-e200038-s001.pdf]

Supplementary Online Content

Courtright KR, Jordan L, Murtaugh CM, et al. Risk factors for long-term mortality and patterns of end-of-life care among Medicare sepsis survivors discharged to home health care. *JAMA Netw Open.* 2020;3(2):e200038. doi:10.1001/jamanetworkopen.2020.0038

eTable 1. Characteristics of Medicare Sepsis Survivor Cohort Discharged to Home Health Care by 1-Year Survival Status

eTable 2. Home Health Assessment Within 1 Week After Sepsis Discharge Among Medicare Beneficiaries by 1-Year Survival Status

eTable 3. Multivariate Regression Results for 1-Year Mortality Among All Sepsis Survivors Stratified by Presence of Cancer

eTable 4. Multivariate Regression Results for Hospice Enrollment Among Decedents Stratified by Presence of Cancer

This supplementary material has been provided by the authors to give readers additional information about their work.

© 2020 Courtright KR et al. *JAMA Network Open.*

eTable 1. Characteristics of Medicare Sepsis Survivor Cohort Discharged to Home Health Care by 1-Year Survival Status

	No. (%)[a]		
**Characteristic**	Died within 365 days of sepsis discharge	Alive 365 days after sepsis discharge	P value
Patients with data	24478 (27.9)	63103 (72.1)	
**Demographics**			
Age			
<65	2808 (11.5)	11428 (18.1)	<.001
65-74	6190 (25.3)	17832 (28.3)	
75-84	7948 (32.5)	20119 (31.9)	
85+	7532 (30.8)	13724 (21.7)	
Race			
Black	2997 (12.6)	7747 (12.7)	<.001
White	19595 (82.3)	49904 (81.5)	
Hispanic	1210 (5.08)	3563 (5.8)	
Other	676 (2.76)	1889 (3.0)	
Female	12626 (51.6)	35846 (56.8)	
Medicaid Eligible	5907 (24.1)	17474 (27.7)	<.001
Annual Family Income, $, mean (standard deviation)	54038 (14902)	54124 (14774)	.44
**Elixhauser comorbidities**			
Hypertension	16217 (66.3)	44257 (70.1)	<.001
Fluid and electrolyte disorders	14021 (57.3)	34418 (54.5)	<.001
Deficiency anemias	10020 (40.9)	21667 (34.3)	<.001
Renal failure	9512 (38.9)	20290 (32.2)	<.001
Alzheimer's disease and related disorders	9488 (38.8)	18607 (29.5)	<.001
Chronic pulmonary disease	8743 (35.7)	19410 (30.8)	<.001
Congestive heart failure	7421 (30.3)	14058 (22.3)	<.001
Diabetes w/o chronic complications	6746 (27.6)	18197 (28.8)	<.001
Metastatic cancer, Lymphoma, Solid Tumor w/o metastasis[b]	5965 (24.4)	5462 (8.7)	<.001
Hypothyroidism	4957 (20.3)	12383 (19.6)	.037
Coagulopathy	4423 (18.1)	9111 (14.4)	<.001
Weight loss	3808 (15.6)	5885 (9.3)	<.001
Other neurological disorders	3530 (14.4)	8428 (13.4)	<.001
Peripheral vascular disease	3236 (13.2)	7233 (11.5)	<.001
Depression	3098 (12.7)	8852 (14.0)	<.001
Obesity	2585 (10.6)	10586 (16.8)	<.001
Diabetes w/ chronic complications	2462 (10.1)	7210 (11.4)	<.001
**Index sepsis hospitalization**			
Infection source			
Kidney, Urinary Tract and Other Genitourinary	10088 (41.2)	26114 (41.4)	.65
Pneumonia and Other Respiratory	9903 (40.5)	20763 (32.9)	<.001
Bone, Joint and Skin/Soft Tissue	3066 (12.5)	10392 (16.5)	<.001
Gastrointestinal	2227 (9.10)	6494 (10.3)	<.001
Device-related	854 (3.5)	2410 (3.8)	.022
Bacteremia	580 (2.4)	1412 (2.2)	.25
Cardiovascular/Endocarditis	297 (1.2)	966 (1.5)	<.001
Postoperative	273 (1.1)	1632 (2.6)	<.001

© 2020 Courtright KR et al. *JAMA Network Open.*

Central Nervous System	52 (0.2)	279 (0.4)	<.001
Other or Unknown	7079 (28.9)	17880 (28.3)	.09
Sepsis severity			
Sepsis	2671 (10.9)	9263 (14.7)	<.001
Severe Sepsis	20365 (83.2)	50148 (79.5)	
Septic Shock	1442 (5.9)	3692 (5.9)	
Intensive/Cardiac care unit admission	12676 (51.8)	31565 (50.0)	<.001
Medical admission type	20969 (85.7)	49166 (77.9)	<.001

[a] Total percentage in each category may not add up to 100 due to rounding

© 2020 Courtright KR et al. *JAMA Network Open.*

eTable 2. Home Health Assessment Within 1 Week After Sepsis Discharge Among Medicare Beneficiaries by 1-Year Survival Status

	No. (%)[a]		
	Died within 365 days of sepsis discharge	Alive 365 days after sepsis discharge	P value
Patients with data	24478 (27.9)	63103 (72.1)	
**OASIS-C Item**			
Indicators for Risk of Hospitalization[b]			
Decline in mental, emotional, behavioral	4851 (19.8)	8574 (13.6)	<.001
Multiple hospitalizations (≥2 in past 12 months)	14329 (58.5%)	29664 (47.0)	
Frailty indicators (weight loss, self-reported exhaustion)	12052 (49.2)	24075 (38.2)	
Assessment of Overall Health Status			
Stable no risks of complications/death	817 (3.3)	3431 (5.4)	<.001
Temporarily high risks but likely to return to stable	8506 (34.7)	33575 (53.2)	
Fragile health/ongoing risks of complications death	11320 (46.2)	22630 (35.9)	
Serious progressive conditions that could lead to death within 1 year	3780 (15.4)	3354 (5.3)	
Unknown/Missing	55 (0.2)	113 (0.2)	
Impaired Vision	17529 (71.6)	48521 (76.9)	<.001
Speech and Oral Expression			
Expresses complex ideas/feelings/needs with no impairment	12113 (49.5)	38717 (61.4)	<.001
Minimal difficulty in expressing ideas and needs	8134 (33.2)	18347 (29.1)	
Expresses simple ideas/needs with moderate difficulty	2394 (9.7)	3769 (6.0)	
Severe difficulty expressing simple ideas/needs	1041 (4.3)	1285 (2.0)	
Unable to express basic needs but not comatose or unresponsive	465 (1.9)	580 (0.9)	
Patient nonresponsive or unable to speak	331 (1.4)	405 (0.6)	
Frequency of Pain Interfering with Activity			
No pain	6755 (27.6)	16352 (25.9)	<.001
Pain that does not interfere with activity or movement	2151 (8.8)	5663 (9.0)	
Less often than daily	2804 (11.5)	7344 (11.6)	
Daily, but not constantly	9792 (40.0)	25819 (40.9)	
All the time	2976 (12.2)	7925 (12.6)	
Living Arrangements			
Lives alone	3765 (15.4)	12639 (20.0)	<.001
Lives with someone	18993 (77.6)	47105 (74.6)	
Lives in congregate (e.g., assisted living)	1720 (7.03)	3359 (5.3)	
Presence of Dyspnea			
Not short of breath	4374 (17.9)	14682 (23.3)	<.001
Walking >20ft, climbing stairs	4176 (17.1)	14291 (22.6)	
With moderate exertion	8074 (33.0)	20913 (33.1)	
With minimal exertion	5765 (23.6)	10428 (16.5)	
At rest (during day or night)	2089 (8.5)	2789 (4.4)	
Respiratory Treatments Needed	8589 (35.1)	15202 (24.1)	<.001

© 2020 Courtright KR et al. *JAMA Network Open.*

Cognitive Function			
Alert/oriented	10764 (44.0)	35285 (55.9)	<.001
Requires prompting	8365 (34.2)	19528 (30.9)	
Requires assistance/direction	3215 (13.1)	5759 (9.1)	
Requires considerable assistance/direction	1555 (6.4)	1867 (3.0)	
Totally dependent	579 (2.37)	664 (1.1)	
Count of ADL/IADL Dependencies			
0-2	1774 (7.3)	8936 (14.2)	<.001
3-5	4583 (18.7)	17734 (28.1)	
6-8	5731 (23.4)	16770 (26.6)	
9-11	8771 (35.8)	15721 (24.9)	
12-13	3619 (14.8)	3942 (6.3)	

[a] Total percentage in each category may not add up to 100 due to rounding
[b] Responses were not mutually exclusive
Abbreviations: ADL/IADL, Activities of Daily Living/Independent Activities of Daily Living

© 2020 Courtright KR et al. *JAMA Network Open.*

eTable 3. Multivariate Regression Results for 1-Year Mortality Among All Sepsis Survivors Stratified by Cancer

	Odds Ratio (95% Confidence Interval; P value)	
**Characteristic**	Cancer (N=11393)	Non-cancer (N=76020)
**Demographics**		
Age (ref=65-74)		
<65	0.94 (0.82, 1.078; .38)	0.84 (0.79, 0.90; <.001)
75-84	0.98 (0.89, 1.08; .70)	1.14 (1.08, 1.20; <.001)
85+	1.09 (0.95, 1.23; .21)	1.58 (1.50, 1.67; <.001)
Race (ref=White)		
Black	1.11 (0.98, 1.26; .11)	1.02 (0.97, 1.09; .42)
Hispanic	0.93 (0.76, 1.13; .46)	0.89 (0.82, 0.97; .008)
Other	0.96 (0.75, 1.22; .72)	0.80 (0.72, 0.90; <.001)
Female	0.88 (0.81, 0.95; .001)	0.83 (0.80, 0.86; <.001)
Medicaid Eligible	0.91 (0.82, 1.02; .1)	0.96 (0.92, 1.00; .07)
**Comorbidities**		
Hypertension	0.905 (0.83, 0.98; .019)	0.86 (0.83, 0.90; <.001)
Fluid and electrolyte disorders	1.01 (0.94, 1.09; .75)	1.04 (1.00, 1.07; .07)
Deficiency anemias	1.21 (1.12, 1.31; <.001)	1.17 (1.13, 1.21; <.001)
Renal failure	1.00 (0.91, 1.09; .93)	1.46 (1.40, 1.51; <.001)
Chronic pulmonary disease	1.06 (0.96, 1.16; .24)	1.23 (1.18, 1.28; <.001)
Diabetes	1.01 (0.93, 1.10; .82)	1.00 (0.96, 1.03; .81)
Congestive heart failure	1.14 (1.03, 1.26; .013)	1.40 (1.34, 1.45; <.001)
Obesity	0.70 (0.61, 0.80; <.001)	0.74 (0.70, 0.78; <.001)
Depression	0.93 (0.82, 1.04; .21)	0.93 (0.88, 0.98; .010)
Peripheral vascular disease	0.92 (0.80, 1.05; .22)	1.26 (1.20, 1.33; <.001)
Weight loss	1.44 (1.23, 1.60; <.001)	1.50 (1.42, 1.58; <.001)
Alzheimer's disease and related disorders	0.75 (0.67, 0.83; <.001)	1.21 (1.15, 1.26; <.001)
**Index sepsis hospitalization**		
Infection source		
Bone/Joint/Skin/Tissue	0.88 (0.77, 0.99; .043)	1.06 (1.01, 1.12; .026)
Central Nervous System	0.73 (0.34, 1.56; .42)	0.72 (0.51, 1.03; .07)
Pneumonia and Other Respiratory	1.04 (0.95, 1.14; .39)	1.17 (1.12, 1.22; <.001)
Postoperative	0.52 (0.41, 0.66; <.001)	0.60 (0.50, 0.71; <.001)
Sepsis severity (ref=Sepsis)		
Severe Sepsis	1.19 (1.06, 1.33; .004)	1.34 (1.27, 1.42; <.001)
Septic Shock	1.16 (0.96, 1.40; .12)	1.14 (1.04, 1.26; .005)
Intensive/Cardiac care unit admission	0.88 (0.81, 0.95; .002)	1.12 (1.08, 1.16; <.001)
Surgical admission type	0.73 (0.66, 0.81; <.001)	0.70 (0.66, 0.73; <.001)
**Home Health Assessment within 7 days of sepsis discharge**		
Indicators for Risk of Hospitalization (ref=No risks)		
Decline in mental, emotional, behavioral	0.99 (0.88, 1.11; .81)	0.99 (0.94, 1.04; .75)
Multiple hospitalizations (≥2 in past 12 months)	1.12 (1.04, 1.22; .005)	1.23 (1.19, 1.28; <.001)
Frailty indicators (weight loss, self-reported exhaustion)	1.10 (1.01, 1.19; .03)	1.06 (1.02, 1.10; .004)
Assessment of Overall Health Status (ref =No risk) [a]		
Temporarily high risks but likely to return to stable	1.01 (0.81, 1.26; .95)	0.95 (0.87, 1.05; .32)
Fragile health/ongoing risks of complications/death	1.65 (1.32, 2.07; <.001)	1.34 (1.22, 1.47; <.001)

© 2020 Courtright KR et al. *JAMA Network Open.*

Serious progressive conditions that could lead to death within 1 year	2.98 (2.33, 3.80; <.001)	1.99 (1.78, 2.22; <.001)
Vision (Ref=No impairment)		
Partially impaired	0.95 (0.86, 1.06; .34)	0.97 (0.93, 1.01; .17)
Severely impaired	0.89 (0.62, 1.29; .54)	0.99 (0.88, 1.11; .85)
Speech and Oral Expression (Ref=No impairment)		
Minimal impairment	1.00 (0.89, 1.11; .95)	1.03 (0.98, 1.08; .25)
Moderate to severe impairment	0.98 (0.80, 1.19; .82)	1.11 (1.03, 1.20; .015)
Unable to speak	1.18 (0.71, 1.98; .52)	1.16 (1.00, 1.34; .05)
Cognitive Function (Ref=No impairment)		
Mild	1.03 (0.93, 1.15; .56)	0.99 (0.94, 1.04; .62)
Moderate	1.25 (0.89, 1.74; .20)	1.14 (1.03, 1.26; .015)
Severe	1.57 (0.81, 3.08; .19)	1.06 (0.90, 1.25; .47)
Living Arrangements (Ref=Lives alone)		
Lives with someone	1.17 (1.05, 1.31; .005)	1.04 (0.99, 1.09; .12)
Lives in congregate (e.g., assisted living)	1.22 (0.94, 1.59; .14)	1.18 (1.09, 1.28; <.001)
Count of ADL/IADL dependencies (Ref=0-2)		
3-5	1.14 (1.00, 1.31; .06)	1.12 (1.04, 1.21; .002)
6-8	1.34 (1.17, 1.54; <.001)	1.35 (1.25, 1.45; <.001)
9-11	1.92 (1.65, 2.23; <.001)	1.94 (1.80, 2.09; <.001)
12-13	2.55 (2.01, 3.23; <.001)	2.91 (2.65, 3.19; <.001)
Presence of Dyspnea (Ref=Not short of breath)		
With moderate exertion / Walking > 20ft, climbing stairs	1.06 (0.95, 1.18; .29)	1.05 (1.01, 1.11; .032)
With minimal exertion	1.24 (1.08, 1.42; .002)	1.31 (1.23, 1.39; <.001)
At rest (during day or night)	1.47 (1.21, 1.80; <.001)	1.54 (1.42, 1.68; <.001)
Frequency of Pain Interfering with Activity (Ref=No pain)		
Sometimes	0.89 (0.79, 1.00; .044)	0.90 (0.86, 0.95; <.001)
Often	1.00 (0.91, 1.11; .94)	0.88 (0.84, 0.92; <.001)
Log Likelihood	-7,245.50	-37754.85
Akaike Information Criterion	14,598.99	75617.69

[a] Patients with an "unknown or unclear" or missing response for Overall Health Status were excluded from regression analyses (N=168)

Abbreviations: ADL/IADL, Activities of Daily Living/Independent Activities of Daily Living

© 2020 Courtright KR et al. *JAMA Network Open.*

eTable 4. Multivariate Regression Results for Hospice Enrollment Among Decedents Stratified by Cancer

	Odds Ratio (95% Confidence Interval; P value)	
**Characteristic**	Cancer (N=5944)	Non-cancer (N=18479)
**Demographics**		
Age (ref=65-74)		
<65	0.83 (0.69, 1.00; .05)	0.76 (0.67, 0.86; <.001)
75-84	1.08 (0.94, 1.23; .28)	1.34 (1.23, 1.46; <.001)
85+	1.06 (0.89, 1.27; .50)	1.64 (1.50, 1.80; <.001)
Race (ref=White)		
Black	0.75 (0.63, 0.89; .001)	0.64 (0.57, 0.70; <.001)
Hispanic	0.90 (0.67, 1.20; .46)	0.77 (0.67, 0.89; <.001)
Other	0.73 (0.52, 1.02; .07)	0.61 (0.50, 0.74; <.001)
Female	1.04 (0.93, 1.17; .49)	1.11 (1.04, 1.18; .001)
Medicaid Eligible	0.77 (0.66, 0.90; .001)	0.78 (0.72, 0.84; <.001)
**Comorbidities**		
Hypertension	0.90 (0.80, 1.02; .09)	0.97 (0.90, 1.03; .29)
Fluid and electrolyte disorders	1.09 (0.97, 1.22; .13)	0.96 (0.90, 1.02; .20)
Deficiency anemias	1.06 (0.95, 1.19; .32)	1.01 (0.95, 1.07; .84)
Renal failure	0.86 (0.76, 0.98; .024)	0.97 (0.91, 1.03; .31)
Chronic pulmonary disease	0.86 (0.76, 0.98; .019)	0.91 (0.85, 0.97; .005)
Diabetes	0.92 (0.81, 1.04; .19)	0.91 (0.85, 0.97; .004)
Congestive heart failure	0.75 (0.66, 0.86; <.001)	0.87 (0.82, 0.93; <.001)
Obesity	0.89 (0.72, 1.09; .25)	0.84 (0.76, 0.93; .001)
Depression	1.21 (1.02, 1.44; .033)	1.11 (1.02, 1.22; .022)
Peripheral vascular disease	0.92 (0.76, 1.12; .39)	0.84 (0.77, 0.92; <.001)
Weight loss	0.94 (0.82, 1.08; .39)	1.10 (1.01, 1.21; .032)
Alzheimer's disease and related disorders	0.84 (0.72, 0.97; .02)	1.14 (1.06, 1.23; <.001)
**Index sepsis hospitalization**		
Infection source		
Bone/Joint/Skin/Tissue	0.76 (0.63, 0.92; .004)	0.80 (0.72, 0.87; <.001)
Central Nervous System	0.48 (0.15, 1.51; .21)	0.93 (0.47, 1.85; .84)
Pneumonia and Other Respiratory	0.86 (0.76, 0.97; .014)	0.97 (0.91, 1.04; .36)
Postoperative	0.97 (0.65, 1.45; .89)	0.97 (0.69, 1.37; .86)
Sepsis severity (ref=Sepsis)		
Severe Sepsis	0.92 (0.77, 1.09; .33)	1.11 (1.00, 1.23; .049)
Septic Shock	0.76 (0.58, 0.98; .038)	0.94 (0.80, 1.11; .47)
Intensive/Cardiac care unit admission	0.84 (0.75, 0.94; .003)	0.94 (0.88, 1.00; .039)
Surgical admission type	1.06 (0.92, 1.23; .44)	0.85 (0.77, 0.93; .001)
**Home Health Assessment within 7 days of sepsis discharge**		
Indicators for Risk of Hospitalization (ref=No risks)		
Decline in mental, emotional, behavioral	1.08 (0.93, 1.27; .32)	1.05 (0.97, 1.14; .27)
Multiple hospitalizations (≥2 in past 12 months)	0.93 (0.83, 1.05; .23)	1.04 (0.97, 1.11; .25)
Frailty indicators (weight loss, self-reported exhaustion)	1.09 (0.97, 1.23; .15)	1.02 (0.96, 1.09; .55)
Assessment of Overall Health Status (ref =No risk) [a]		
Temporarily high risks but likely to return to stable	1.24 (0.87, 1.77; .24)	0.98 (0.83, 1.16; .81)
Fragile health/ongoing risks of complications/death	1.65 (1.16, 2.35; .006)	1.14 (0.96, 1.35; .13)

© 2020 Courtright KR et al. *JAMA Network Open.*

Serious progressive conditions that could lead to death within 1 year	1.97 (1.37, 2.85; <.001)	1.240 (1.03, 1.50; .024)
Vision (Ref=No impairment)		
Partially impaired	1.07 (0.92, 1.24; .37)	0.96 (0.90, 1.03; .29)
Severely impaired	1.04 (0.64, 1.69; .88)	1.08 (0.90, 1.30; .41)
Speech and Oral Expression (Ref=No impairment)		
Minimal impairment	1.01 (0.87, 1.17; .91)	1.02 (0.93, 1.11; .73)
Moderate to severe impairment	0.86 (0.67, 1.12; .26)	1.17 (1.03, 1.32; .013)
Unable to speak	0.82 (0.46, 1.45; .49)	0.98 (0.79, 1.22; .86)
Cognitive Function (Ref=No impairment)		
Mild	1.05 (0.90, 1.21; .55)	1.08 (0.99, 1.17; .09)
Moderate	1.30 (0.86, 1.97; .22)	1.11 (0.94, 1.31; .21)
Severe	0.41 (0.20, 0.81; .010)	0.83 (0.65, 1.07; .14)
Living Arrangements (Ref=Lives alone)		
Lives with someone	1.01 (0.86, 1.19; .92)	1.20 (1.10, 1.31; <.001)
Lives in congregate (e.g., assisted living)	1.66 (1.11, 2.50; .014)	1.95 (1.69, 2.23; <.001)
Count of ADL/IADL dependencies (Ref=0-2)		
3-5	1.21 (0.97, 1.50; .09)	1.18 (1.03, 1.36; .02)
6-8	1.09 (0.87, 1.35; .46)	1.21 (1.05, 1.39; .007)
9-11	1.09 (0.87, 1.37; .46)	1.29 (1.12, 1.48; <.001)
12-13	1.11 (0.82, 1.52; .49)	1.20 (1.01, 1.41; .034)
Presence of Dyspnea (Ref=Not short of breath)		
With moderate exertion / Walking > 20ft, climbing stairs	0.82 (0.70, 0.97; .019)	0.99 (0.91, 1.08; .84)
With minimal exertion	0.80 (0.66, 0.97; .026)	1.00 (0.90, 1.11; .95)
At rest (during day or night)	0.70 (0.55, 0.91; .006)	0.94 (0.82, 1.08; .39)
Frequency of Pain Interfering with Activity (Ref=No pain)		
Sometimes	1.07 (0.91, 1.27; .42)	0.98 (0.90, 1.07; .63)
Often	1.05 (0.91, 1.21; .50)	0.96 (0.89, 1.04; .31)
Log Likelihood	-3693.626	-12048.21
Akaike Information Criterion	7495.251	24204.42

© 2020 Courtright KR et al. *JAMA Network Open.*
